# Development of an interview practice course for improving overall confidence in specialty training national selection in vascular and general surgery

**DOI:** 10.1016/j.sopen.2025.04.004

**Published:** 2025-04-17

**Authors:** Melvin Joy, Wen Ling Choong, ChangShi Tang, Marta Madurska, Benjie Tang, Brian Ip

**Affiliations:** a Department of General Surgery, Ninewells Hospital and Medical School, Dundee DD1 9SY, United Kingdom; b Einburgh School of Medicine, University of Edinburgh, Edinburgh EH8 9YL, United Kingdom; c R Adams Cowley Shock Trauma Center, University of Maryland Medical System, MD, USA; d Surgical Skills Centre, Respiratory Medicine and Gastroenterology, School of Medicine, University of Dundee, Dundee DD1 9SY, United Kingdom; e Department of General Surgery, Dumfries Royal Infirmary, Cargenbridge DG2 8RX, United Kingdom

**Keywords:** Peer-delivered learning, Patient communication skills, Technical skills and teaching, Specialty training national selection, Surgery

## Abstract

**Objectives:**

To evaluate the effectiveness of a training course to improve surgical trainees' confidence in specialty training national selection (STNS) for higher surgical training in surgery. It was also the aim to identify weak areas in the current two-year core surgical training programme in the United Kingdom.

**Methods:**

A prospective observational study was conducted. Delegates were asked to complete evaluation forms to track their perceived confidence levels of success in STNS at different timeframes, measured by a visual analogue scale.

**Setting:**

The 2-day interview preparation course was designed with a maximum delegate number of ten per course in Surgical Skills Centre, Ninewells Hospital, University of Dundee, UK.

**Participants:**

Twenty-seven delegates provided feedback of their perceived confidence levels of success at STNS higher surgical training in general and vascular surgery.

**Results:**

Delegate self-reported confidence increased significantly for all domains except Patient Communication (6.12 ± (1.75) vs 7.10 ± (1.69), P = 0.063). A lower confidence was reported by UK graduates and first-time applicants in the technical and teaching domain (6.03 ± 2.04 vs 7.24 ± 1.92, p = 0.007). 23 (85 %) of the participants were successful in the STNS post course.

**Conclusions:**

Peer-delivered teaching, practice and feedback as a structured interview practice course can significantly improve applicants' overall confidence levels in preparing for STNS and a high success rate at STNS. Patient communication skills training and education should be enhanced in the training programme. A lower confidence was reported by UK graduates and first-time applicants in the technical and teaching domain.

## Introduction

Appointment to higher surgical training in the UK from Core Surgical Training is competitive. Since 2011, eligible Core Surgical Trainees (or equivalent) wishing to obtain Certificate of Complete Training (CCT) in General or Vascular Surgery undergo a 3-h structured interview at a National Selection (NS) Centre. The interview is hosted by Health Education England (HEE) on behalf of the Vascular Surgery and General Surgery Specialty Advisory Committees (SAC) [1]. The structure of the Specialty Training (ST3) General and Vascular Surgery Interview puts emphasis on 6 different domains: (i) Clinical Scenario, (ii) Clinical Management, (iii) Patient Communication, (iv) Portfolio, (v) Academic Knowledge, (vi) Teaching and Technical Skills. Simulated cases based on each assessed domain are designed to closely mirror real life surgical practice. Candidates who have achieved an overall score which exceeds a defined threshold are deemed appointable. Competition ratio has steadily decreased from 3.32:1 in 2012 to 1.48:1 in 2017 [2]. The junior doctors' contract dispute of 2016 [3] led to a disproportionate drop in applicant numbers. This reduction was against a small increase in the number of training posts. Despite improved statistics, many applicants were deemed ‘unappointable’ by score. Whilst such observation may be explained by lack of real-life clinical experience, many candidates anecdotally cite poor interview technique as a cause [4].

We hypothesised that the deficiencies and incompetence of certain key elements of clinical and surgical skills led to the unsuccessful interview of selection process. Identification and reinforcement of training in the weak areas will improve the quality of core surgical training. To improve confidence and be effective; therefore, we developed a structured we developed a structured peer-delivered interview preparation course to address these barriers [5].

The aims of this study were to: 1) evaluate the effectiveness of our course in improving the self-reported confidence of the applicants at the UK ST3 General and Vascular Surgery Interview; 2) identify the weak areas of knowledge and skills of core surgical trainees in the current surgical training programme utilised in the UK; and 3) evaluate the impact on the success rate of NS of the course on non-UK medical graduates and non-first-time applicants.

## Materials and methods

Prospective applicants who intended to apply to the UK ST3 General and Vascular Interview applied voluntarily to attend the interview training course. The course was advertised online, indirectly via social media (e.g. Twitter), the Association of Surgeons in Training webpage, and the Royal College of Surgeons of Edinburgh's ‘Surgeons News’ publication. The delegates were self-selected to attend the course from various background. The delegates came from all over the Uk where some were trained in general teaching hospitals while other were trained in district hospitals. There was no selection criteria imposed to the delegates by the either the course organizer or faculty. The course fee was paid for the venue hire, facilities, consumables, food, models for OSCE stations. There was no financial gain for the faculty.

The course was designed based on initial feedback from a focus group of 4 surgical trainees who were appointed in the preceding 12 months. A Delphi consensus processes were conducted before the course. The Delphi processes were initiated by identifying the number, credentials and experience of the potential faculty, course format and duration, and delegate to faculty ratio. The processes were conducted by 2 general consultant surgeons and 8 ST3-ST8 surgical trainees who had been examiners in the interview process in General and Vascular Surgery. It was agreed that course faculty should consist of General Surgery and Vascular Surgery trainees who were at level at ST3 to ST8 in general and vascular Surgery. The course was designed based on the forementioned Delphi process. Based on the experience and feedback gained from the pilot study, the second Delphi consensus process was conducted, and the consensus were used to refine the design of the course.

**Fig. 1:**
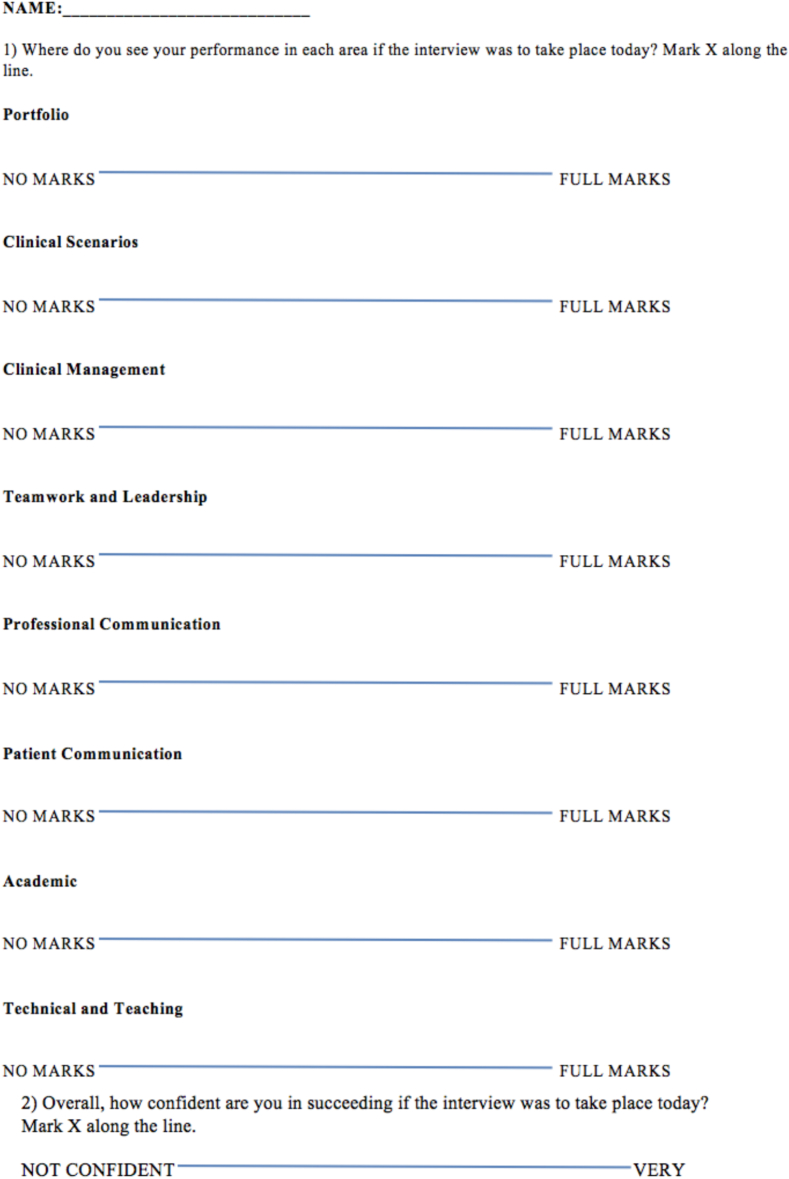
Course programme.

The 2-day interview preparation course was designed with a maximum delegate number of ten per course (Fig. 1). The delegate to tutor ratio was kept at one to one. Each delegate was assigned a mentor at the beginning of the course to serve as a single point of contact over the entire course. In addition, surgical consultants' presence ensured clinical accuracy and maintained credibility. Faculty were briefed before the course on giving feedback in a standardised manner.

The first day began with didactic lectures focusing on each domain within the interview process: Portfolio, Clinical Skills, Clinical Management, Patient Communication skills, Academic, Technical Skills and Teaching. This was followed by moulage which allowed for practice and immediate feedback on performance. The second half of day one involved peer-delivered teaching and practice on selected technical skills. Delegates worked in pairs, allowing them to observe each other's performance. In addition to verbal feedback, each delegate was scored individually using a structured scoring sheet with scores of 1–5. This score sheet mirrored the one used in the real-life interview.

Day two began with an Objective Structured Clinical Examination (OSCE) simulating the entire interview process. OSCEs have been used since the 1970s in medical education to prepare healthcare students for real clinical practice in a safe environment. OSCEs allowed application and reinforcement of theoretical learning and provided real-time feedback on areas for improvement [6]. By emphasising a safe, non-judgemental learning environment, delegates reframe their mindset to overcome their anxiety barrier. This is due to the perception that “the stakes are not as high” in the mock environment [7,8].

The Procedural Based Assessment (PBA) was used to assess the 6 main domains of skills in the current study. The PBA was originally invented and validated by the Orthopaedic Competence Assessment Project (OCAP) for Trauma and Orthopaedic Surgery and was modified by the Specialty Advisory Committees for surgery for the assessment of surgical trainees in all the surgical specialties. The PBA was used to assess surgical trainee's technical, operative and professional skills in a range of specialty procedures or parts of procedures [9]. Delegates rotated individually through stations representing each of the assessed domains, manned by one faculty member who delivered immediate verbal feedback as well as written feedback.

First, a group faculty reviewed and discussed all scoring sheets of delegates, then the meeting for feedback was led by the delegate's mentor. Led by the delegate's mentor, individualised, constructive formative feedback was delivered with contributions from the rest of the faculty. Specific areas of development were identified based on the consensus of the faculty supported by the data from the scoring sheets. Following this a second OSCE run was conducted with written scoring and no verbal feedback to simulate the actual interview process. The delegate was given the opportunity to review all collated personalised feedback at the end of the course. Qualitative feedback was provided from the course delegates after the course.

There were no patients or other conflicting materials involved in this study. The ethical committee advised that the consent from the participants should be sufficient for the ethical approval because of the nature of the study. Delegates were informed clearly that the participation of the study was purely voluntary. Consent forms were obtained from the delegates. All delegates consented to their data being used in anonymised manner for the purpose of this study.

**Fig. 2:**
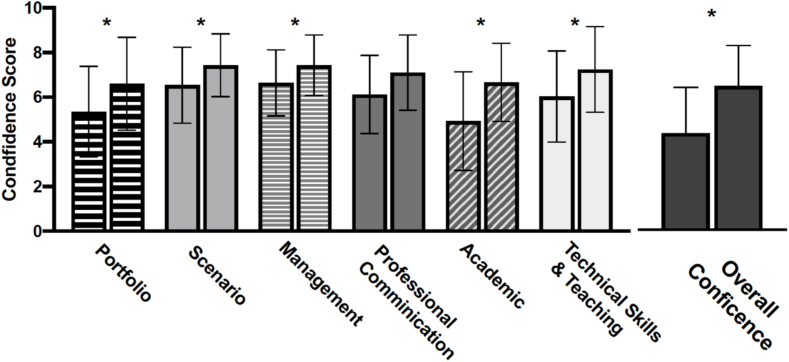
Visual analogue scale for self-assessment of confidence.

A pre-course evaluation form was used to collect delegate demographic data including current level of training, duration of preparation, methods of preparation, number of previous ST3 interview attempts as well as self-evaluation using a visual analog scale (VAS) on perceived confidence level of success in NS. VAS was chosen as a self-assessment tool given its ease of use, reliability and the ability to assess change over time [10]. No confidence was scored as 0 cm while total confidence was scored as10 cm on VAS scoring. The evaluation consisted of a subjective assessment of the participants overall confidence in performance in the interview as well as confidence in each of the specific components: Portfolio, Clinical Scenario, Management, Patient Communication, Academic, Technical and Teaching Skills. Delegates scored themselves by placing an ‘X’ along the VAS line to denote their perceived level of confidence, ranging from low to high (Fig. 2). Each line measured 10 cm with one end being “no marks/not confident” and the other end, “full marks/very confident”. Data on confidence were gathered using the VAS tool pre- and post-course. Data was collated per delegate by measuring in centimetres to one decimal point to assess the level of confidence and compare progression of this parameter through the course.

Statistical analysis was performed using the SPSS Statistical program for Mac version 26 (IBM, Chicago, Ill). Normality testing was performed to determine data distribution. As data showed a normal distribution, continuous data is presented using mean and standard deviation and categorical data is presented using percent. Paired samples *t*-test was performed to compare means before and after the course completion. Statistical significance was set at p value of <0.05.

## Results

Twenty-seven delegates completed the full 2-day course and submitted evaluations. Demographic data is presented in Table 1. 44 % of the course participants were at Core Trainee (CT) level, 30 % were at level of Locum in Training (LAT) ST3, with the remaining participants (26 %) above ST3 level (including Locum in Service (LAS), ST, clinical fellow posts or others). 56 % of participants have not attempted the interview before, 30 % had one previous failed attempt, and 14 % had >1 failed attempt [2,3]. Non-UK graduates accounted for 37 % of the cohort.

**Table 1:** Participant demographics.

Participants	N	%
*Level of training*		
CT2	12	44 %
LAT ST3	8	30 %
Above of ST3 equivalent	7	26 %

*Number of previous attempts*		
0	15	56 %
1	8	30 %
>1	4	14 %

**Fig. 3:**
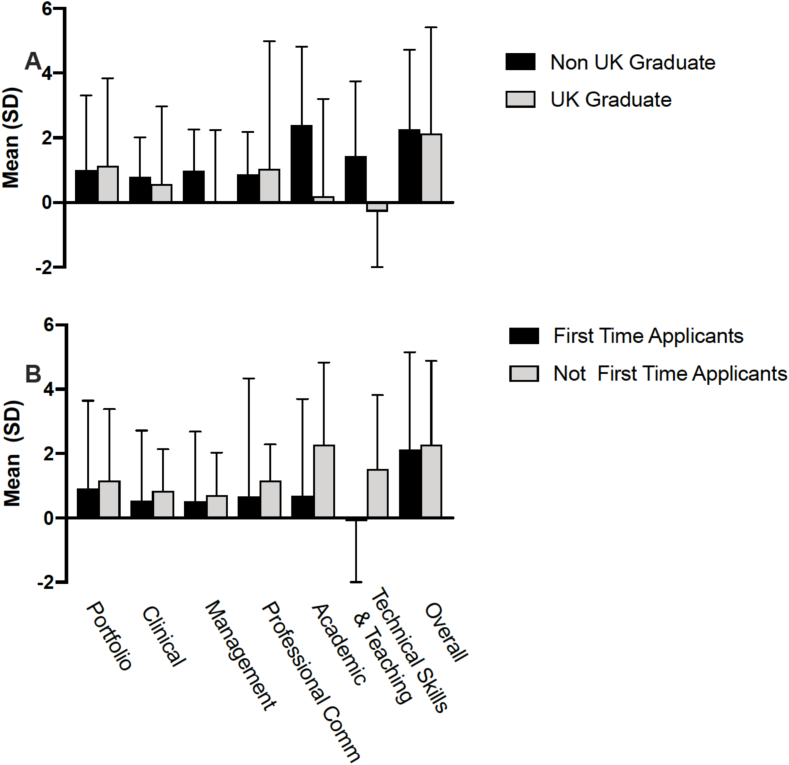
Graphical illustration of the level of confidence before and after the course.

Following the course, delegate self-reported confidence increased significantly. The Portfolio station was improved noticeably (5.35 ± 2.03 vs 6.60 ± 2.08, p = 0.008). Confidence score was also improved after completion of both Clinical scenario (6.54 ± 1.70 vs 7.43 ± 1.41, p = 0.004) and Management stations, (6.64 ± 1.48 vs 7.43 ± 1.36, p = 0.012). Similarly, there were improvements in confidence in the Technical and Teaching Skills station (6.03 ± 2.04 vs 7.24 ± 1.92, p = 0.007) as well as the Academic station (4.93 ± 2.21 vs 6.66 ± 1.75, p = 0.004). There was no overall increase in confidence in either the Patient Communication station (6.12 ± 1.75 vs 7.10 ± 1.69, p = 0.063) or Technical Skills & Teaching (6.03 ± 2.04 vs 7.24 ± 1.92, P = 0.07). The UK graduates and first-time applicants had lower confidence in the technical and teaching domain. Overall confidence of success at National Selection increased significantly (4.32 ± 2.05 vs 6.43 ± 1.81, p < 0.001) (Table 2 and Fig. 3). Non-UK graduates and non-first-time applicants reported more experience in carrying out or assisting in these procedures in their feedback.

**Table 2:** Levels of confidence before and after the course.

Station	Before Mean (SD)	After Mean (SD)	p
Portfolio	5.35 (2.03)	6.60 (2.08)	0.008
Clinical scenario	6.54 (1.70)	7.43 (1.41)	0.004
Clinical management	6.64 (1.48)	7.43 (1.36)	0.012
Patients communication	6.12 (1.75)	7.10 (1.69)	0.063
Academic	4.93 (2.21)	6.66 (1.75)	0.004
Technical skills & teaching	6.03 (2.04)	7.24 (1.92)	0.07
***Overall confidence***	4.32 (2.05)	6.43 (1.81)	<0.001

**Fig. 4:**
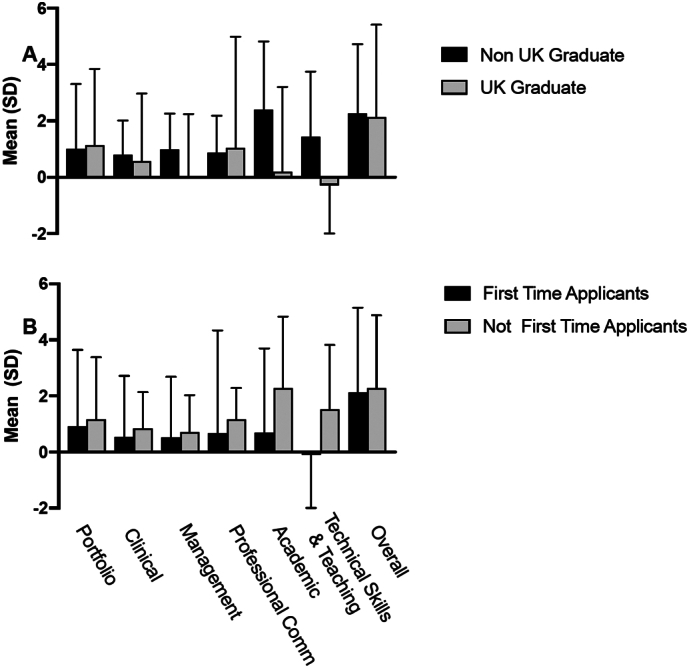
Difference in confidence score before and after the course presented in mean and standard deviation (SD) for each station as well as overall course. Comparison between: A. Non-UK Graduate and UK Graduate course participants; B. participants who were first time applicants and those who have completed the recruitment process at least once before. P > 0.05 for each category.

The change in overall confidence did not differ between UK graduates and non- UK graduates (1.91 ± 0.54 vs 2.08 ± 0.67, P = 0.538), nor between first time and not-first-time applicants (1.89 ± 0.43 vs 2.06 ± 0.35, P = 0.893) (Fig. 4A and B). 85 % of the course participants were successful in obtaining a STNS at the interview following the course. The success rates to secure a NS number were 70 % for non-UK graduates compared average NS rater at 43 % and 67 % compared to 21 % for non-first-time applicants respectively [2,11].

## Discussion

In this study, it has been demonstrated that peer-delivered teaching, practice and feedback translated to increased confidence levels as perceived by the delegates. Patient communication skills have been identified as skills that required longer practice, this was demonstrated in the feedback amongst all candidates, particularly, the non-UK graduates struggled to communicate their knowledge effectively in a time-limited, pressurised environment [12]. Patient communication skills should be emphasized throughout training that should be beyond the duration of the 2-day course. These delegates as self-selected cohort attended the course from a wide range of background, therefore, their performance in this area would reflect the reality of their skills and ability in the workplace. To improve patient communication skills, some authors have advocated that communication skills should be taught formally especially to healthcare professionals [13,14]. Based on the data of this survey, we would like to advocate that there should be more attention and effort to be made to improve the training in this area in the core surgical training curriculum. A different type of practice method or teaching could be beneficial for trainees, including improved language proficiency overall which could be best served with other methods than a mock patient interaction for some trainees. The biggest increase was observed in the academic station. Anecdotal feedback from delegates suggested this station represented the greatest psychological barrier due to its perceived complexity and knowledge gap.

A lower confidence was reported by UK graduates and first-time applicants in the technical and teaching domain. Under the technical and teaching stations, five technical skills were selected based on previous application in real life interview: excision of skin lesion, small bowel resection, basic laparoscopy, arteriotomy and vein patch. Assessment of these skills in the course, as in simulated or real-life training exercises, were carried out using Procedural Based Assessments (PBAs). Despite PBAs being readily accessible by surgical trainees on the Intercollegiate Surgical Curriculum Project (ISCP) portal, very few delegates took the effort to revise PBAs prior to the course. Non-UK graduates and non-first-time applicants reported more experience in carrying out or assisting in these procedures, therefore putting them at a higher baseline for confidence and learning. In contrast, UK-graduates and first-time applicants may have felt overwhelmed and alienated by the knowledge and technical skills required to perform a simulated task in a limited timeframe for preparation. Therefore, technical and teaching domain has been identified as a weak area for the core surgical trainees in the current core surgical training programme and there was a significant need to enhance and improve training in this domain.

The concept for this course resulted from informal ‘interview practice’ sessions with many non-UK graduates and non-first-time applicants. There was a perception amongst these groups that they are disadvantaged by individual factors [15]. Our course achieved a 70 % success rate to obtain a National Training Number (NTN) for non-UK graduates. For most non-UK graduates, achieving a NTN provided the most reliable pathway to CCT and independent consultant practice. The current alternative to Specialist Registration (‘Article 14’/CESR) is widely known to be a frustrating process [16].

Good qualitative feedback from the course delegates was consistently received. Improved confidence achieved at the course translated to success at NS rate – 85 % of course delegates obtained training posts compared to the overall successful rate of NS was 67 % [2] in general or vascular Surgery in the UK. Non-first-time applicants are hampered by memories of their previous performance failure. They often find it difficult to part with their negative mentality to succeed. However, we found that there was no statistically significant difference between first time and non-first-time applicants in overall confidence after having completed the training course, this might imply that a well-designed and run training course significantly improved their confidence. Our course achieved a 67 % success rate for non-first-time applicants.

Our course utilised OSCE style learning with minimal lecture-based teaching. Currently, numerous interview preparation courses of varying formats are available in the UK. Although most courses cover similar content, they lack the practical aspect of OSCE style interviews as well as one-to-one peer mentoring during and after the course. As these teaching methods are resource intensive, most of these courses run in a pure didactic lecture format. Such passive format failed to assess the candidates understanding with little personalised feedback. The immediate verbal feedback after the OSCE style interview on the first day as well as written feedback throughout both days about the candidates' performance has been proven a very effective way of benefiting delegates learning. Feedback from a different faculty member with contributions from the rest of the faculty on the 2nd day provided delegates opportunities for multiple assessors, the delegate was given the opportunity to review all collated personalised feedback at the end of the course, which might decrease the likelihood of bias. Delegates are given opportunities to reflect on their strengths and weakness throughout the course. Based on the data generated and experience learned from this study, we believe that the quality of clinical and surgical skills of core surgical training will be enhanced and improved by incorporating OSCE type in to core surgical training programme.

There were some limitations of our study that included the small number of cohorts of 27 delegates and the possible selection bias. Confidence scores were only self-reported, with no objective scoring to correlate with the observed positive trend in confidence levels. There was a lack of information in the relationship between post-course confidence levels and success rates of obtaining an NTN. Investigating whether higher post-course confidence correlated with a higher likelihood of obtaining an NTN would provide stronger evidence of the course's impact. The delegates were a self-selected group, suggesting a degree of insight and motivation in their preparation approach. We were aware that fatigue experienced by delegates during the 2-day course might also influence their self-reported scores. We also were aware that participants attended a paid course, thus, individual financial background and motivation might have introduced a bias. However, the course fee was spent on the cost to cover food, venue hire, OSCE station consumables while there was no financial return for the faculty. Finally, the authors understood that this study only assessed confidence which is subjective. Therefore, an improvement to the methodology would be to compare objective performance score provided by the interviewer in each station to the delegates' confidence scores, this will be addressed in the future study.

## Conclusion

Peer-delivered teaching, practice and feedback as a structured interview practice course can significantly improve applicants' overall confidence levels in preparing for ST3 NS and a high success rate at NS. A lower confidence was reported by UK graduates and first-time applicants in the technical and teaching domain; thus, effort should be made to enhance this domain for the surgical trainees in the current core surgical training programme. Patient communication skills training and education should be enhanced in the training programme.

## CRediT authorship contribution statement

**Melvin Joy:** Writing – original draft, Project administration, Methodology, Investigation, Formal analysis, Data curation, Conceptualization. **Wen Ling Choong:** Writing – original draft, Validation, Methodology, Investigation, Formal analysis, Data curation, Conceptualization. **ChangShi Tang:** Writing – review & editing. **Marta Madurska:** Validation, Project administration, Methodology, Investigation, Data curation, Conceptualization. **Benjie Tang:** Writing – review & editing, Validation, Supervision, Resources, Project administration, Methodology, Investigation, Formal analysis, Data curation, Conceptualization. **Brian Ip:** Writing – review & editing, Supervision, Resources, Project administration, Methodology, Investigation, Formal analysis, Data curation, Conceptualization.

## Ethics approval and consent to participate

There was no patient and other conflicting materials involved in this study. The ethical committee advised that the consent from the participants should be sufficient for the ethical approval because of the nature of the study. Delegates were informed clearly that the participation of the study was purely voluntary and verbal consent was given by the surgical trainees who participated in this study.

## Funding/support statement

This research has been funded by the 10.13039/501100000266Engineering and Physical Sciences Research Council (EPSRC) of the United Kingdom under Grant Reference EP/Y017307/1.

## Declaration of competing interest

All authors declare no competing interests.

## Data Availability

The dataset analysed during the current study is available from the corresponding author on request.
